# The Organisation of the Leicester Saturday Society

**Published:** 1917-10-06

**Authors:** 


### The Organisation of the Leicester Saturday Society.

### SIR EDWARD WOOD'S WORK.

The great influence of the late Sir Edward Wood in the work of the Leicester Royal Infirmary was recorded in The Hospital of last week. The Leicester and County Saturday Hospital Society, of which he was the founder and the president from the time of its registration, is a monument to his initiative, enthusiasm, and enterprise. When he accepted the chairmanship of the Infirmary Sir Edward Wood found that the total contributions from workpeople were £4,086 only, made up of sums taken at annual collections in factories and warehouses. With the foresight which his successful business training gave him, Sir Edward saw that if these spasmodic contributions could be organised into systematic weekly contributions of small amount the return would be greatly increased.

But a scheme had first to be prepared, and in the planning the "builder wrought even better than he knew." First, the Infirmary must be brought into a state of modernity; and, secondly, a convalescent home must be acquired to which workers might be sent for convalescence. Both plans eventuated, the first as described in last week's Hospital, the second in the acquisition of Desford Hall and grounds of 50 acres as a convalescent home, secured by means of the Queen Victoria Memorial Fund raised in the mayoralty of Alderman Lennard, Sir Edward Wood's son-in-law. On the former's sudden death during his mayoralty, Sir Edward Wood undertook his son-in-law's mayoral duties. Acquainted with the systematic collections of the Birmingham Saturday Hospital Society and its great work in the maintenance of convalescent homes for the workers in Wales, Sir Edward Wood invited twenty representative Leicester working men to spend the week-end as his guests at Llandudno, so that they might inspect the homes there. On their return a public meeting was called, when an account of the Birmingham Society's work was submitted and a similar fund was outlined for Leicester. The scheme was heartily endorsed by the Trades Council, Mr. H. H. Woolley was appointed organising secretary, and systematic weekly collections were instituted in factories and workshops, with the result that the income of the Society was quadrupled. Some years later, largely through a bequest from Mr. Edward Higgs, a Leicester citizen, a second home was erected for wives of members and female members of the Society.

"Burdett's Hospitals and Charities" records the fact that Leicester holds pride of place in the amount of the workpeople's contributions per thousand of the inhabitants. In 1916 the Society's income was just under £17,000, allocated as to seven-tenths to the Leicester Royal Infirmary and the remainder to the maintenance of the convalescent homes, etc. The last annual report of the Society disclosed a satisfactory financial condition, £7,000 having been accumulated out of income as a reserve fund.

This successful administration stands largely to the credit of Sir Edward Wood, who in this, as in other matters relating to the work of the town, showed that his vision and outlook were more extensive than those of most people, and his courage of that confident and public-spirited kind which invariably achieves results and proves the great administrator.

